# Tubercular Synovitis and Metastatic Inflammation of the Joints

**Published:** 1897-10

**Authors:** 


TUBERCULAR SYNOVITIS AND METASTATIC INFLAMMATION OF THE JOINTS. From Prof. E. Hess' *Annual Report*.—According to our clinical, pathologico-anatomical and experimental investigations, cases of more or less acute tubercular synovitis occur very often in the elbows, in the joint of the hock, and in the membrane (or sheath) of the muscle extensor digitorum pedis longus in cattle a few weeks after calving, and especially after abortion. We believe, after careful observation, that we are justified in assuming that tuberculosis is the most frequent cause of the so-called inflammation of the tarsal-joint in cattle. It is also certain that lighter forms of tubercular synovitis, with good care and proper treatment, can be cured after weeks or months; nevertheless, for financial reasons, it is better to kill all those animals in which the disease sets in with great severity, remains for a long time unchanged, or steadily grows worse. In connection with this should be mentioned those metastatic inflammations of the joints which represent secondary diseases—the so-called secretory metastasis. These occur not infrequently among our larger and smaller domestic animals, and are often still wrongly diagnosticated as rheumatism of the joints. They are undesirable chemical complications as well as of great importance, from a pathologico-anatomical point of view, in many infectious and febrile diseases, as, *e. g.*, pyæmia, septicæmia, glanders, retentio placentarum, metritis purulenta, vaginitis traumatica, mastitis, acute omphalitis, infectious agalactia in goats, chronic swine-erysipelas and swine-pest, and epilepsy in dogs. On the ground of anatomical investigation we cannot share the opinion, often expressed, that rheumatism of the joints in cattle rests upon an infection arising from a puerperal uterus. On the contrary, we are convinced that those inflammations of the joints, and especially those of the hock-joint—*i. e.*, the so-called udder-inflammation of the joints, which often arises after a normal birth, after abortion, retentio placentarum, metritis, and vaginitis—have nothing to do with rheumatism of the joints, and are really to be regarded as secretory metastasis or tuberculosis. Secretory metastasis bears to the diseases of the genital organs a relation analogous to that of gonorrhœa in man to the gonorrhœal inflammation of the knee-joint.—*Idem.*

